# Clone wars: the evolution of therapeutic resistance in cancer

**DOI:** 10.1093/emph/eow015

**Published:** 2016-06-14

**Authors:** Catherine M. Worsley, Elizabeth S. Mayne, Rob B. Veale

**Affiliations:** 1 Department of Molecular Medicine and Haematology, Faculty of Health Science, University of the Witwatersrand, Johannesburg, South Africa;; 2 Department of Molecular Medicine and Haematology, Charlotte Maxeke Johannesburg Academic Hospital, National Health Laboratory Service, Johannesburg, South Africa;; 3 School of Molecular and Cell Biology, Faculty of Science, University of the Witwatersrand, Johannesburg, South Africa

## THE FAILURE OF CHEMOTHERAPY

Cancer chemotherapy kills some tumour cells, leaving behind resistant clones with less competition for space and resources. These clones, groups of cells that share common ancestry, proliferate without restriction causing disease relapse.

Tumours contain a host of cancer clones that are genetically and epigenetically different from one another [1]. These clones follow a Darwinian process of somatic selection where they compete for space and resources within their microenvironments [2]. Cancer cells acquire mutations over time that affect their fitness, giving some clones a survival advantage over others [3].

Chemotherapy itself is a selective pressure that influences tumour heterogeneity and clonal evolution. Most cancer deaths are caused by clones that are therapeutically resistant [3]. The evolution of resistant clones occurs rapidly, resulting in the appearance of new clones that are not susceptible to conventional therapy. Evolutionary principles describe the process of clonal evolution and aid in formulating novel strategies for disease management and prognosis [4].

## EVOLUTIONARY PERSPECTIVES

An understanding of tumour heterogeneity and clone fitness is key to developing better treatment options. Intra-tumoural genetic variability and instability affect the process of somatic evolution [4].

Variations in the tumour microenvironment, including nutrient availability and blood supply access, drive clone evolution in both the presence and absence of chemotherapeutic drugs [3, 5]. The more genetic or environmental variation there is, the greater the likelihood that some clones will develop a survival advantage over others [4].

In some instances, resistant clones can co-operate with one another in ways that promote their survival [6], leading to faster cancer progression or increased aggressiveness. When chemo-sensitive cells are killed, space and resources become abundantly available to resistant clones, which then proliferate without inhibition by neighbouring cells. Understanding clonal evolution and the pressures that select for resistant clones can inform the development of new therapeutic approaches.

## FUTURE IMPLICATIONS

With the advent of molecular data and computational frameworks, alternate strategies are being investigated to manipulate the microenvironment to control and contain tumours [5, 7]. Genetic profiling of tumour progression over time allows analysis of DNA methylation patterns and base pair mutations of clones, linking the evolution of clones to specific genetic events [8]. Single cell analysis helps in understanding tumour progression and intra-tumoural heterogeneity [9], and next-generation sequencing uncovers genetic complexities in individual clones [3, 10].

Mathematical and computational modelling provides frameworks for determining vital mutations and microenvironmental changes, bridging the gap between laboratory data and clinical information [7, 9, 11].

The strategies being investigated include using cell competition to control resistant clones, manipulating blood and nutrient supply, and using drugs that contain tumour growth instead of killing cells [7, 8, 11]. Many of these approaches are being tested in animal models, with the hope that some of these interventions will make their way into clinical practice.

One promising approach is to utilize tradeoffs by manipulating clones to compromise themselves by becoming dependent on mutations that protect them from one drug, but that make them susceptible to other drugs [12].
